# Supplementary material for: Therapy-Resistant Acute Myeloid Leukemia Stem Cells Are Resensitized to Venetoclax + Azacitidine by Targeting Fatty Acid Desaturases 1 and 2
Source: Metabolites. 2023 Mar 24;13(4):467. doi: 10.3390/metabo13040467 (PMC10142983; doi:10.3390/metabo13040467)
Supplement: Supplementary file 1 [file metabolites-13-00467-s001.zip › Supplementary_032323.pdf]

Therapy resistant acute myeloid leukemia stem cells are resensitized to venetoclax + azacitidine by targeting fatty acid desaturases 1 and 2: Supplementary Materials

Supplementary Methods

Human Specimens:
AML specimens were obtained from apheresis product, peripheral blood, or bone marrow from AML patients, or mobilized peripheral blood from healthy donors who gave informed consent for sample procurement on the University of Colorado tissue procurement protocol. For age, sex, blast percentages, cytogenetic, and mutational information see Supplementary Table S1. Samples defined as "diagnosis" were obtained from patients who had not yet received treatment after being diagnosed. Samples defined as "relapsed/refractory" were obtained from patients who had relapsed after receiving 7+3 chemotherapy, and who are refractory to venetoclax.

Global UHPLC-MS Metabolomics:
Commercial reagents were purchased from Sigma-Aldrich. (Saint Louis, MO) Approximately 100,000-200,000 ROS-Low LSCs were sorted, and analyses were performed via ultra-high pressure-liquid chromatography-mass spectrometry (UHPLC-MS – Vanquish and Q Exactive, Thermo Fisher, San Jose, CA, and Bremen, Germany) using C18 reversed-phase chromatography with separate runs in positive and negative mode, using electrospray ionization (ESI). Briefly, cells were extracted in ice-cold methanol:acetonitrile:water (5:3:2 v/v) at a concentration of 2 million cells/mL. After vortexing for 30 min at 4°C, samples were centrifuged at 12,000 g for 10 min at 4°C. Ten microliters of sample extracts were loaded onto a Kinetex XB-C18 column (150 × 2.1mm i.d., 1.7µm, equipped with a SecurityGuard™ Ultracartridge – UHPLC C18 for 2.1 mm ID columns – Phenomenex, Torrance, CA) at 45°C using a 5 min gradient at 450uL/min. (Positive mode: 5-95% B, A: water/0.1% formic acid, B: acetonitrile/0.1% formic acid; Negative Mode: 0-100% B, A: 5% ACN, 95% water, 1 mM ammonium acetate, B: 95% ACN, 5% water, 1mM ammonium acetate) were used to elute metabolites. The mass spectrometer was scanned in Full MS mode at 70,000 resolution in the 65-975 m/z range, 4 kV spray voltage, 45 sheath gas, and 15 auxiliary gas, operated in negative and then positive ion mode (separate runs). Calibration was performed before analysis using the PierceTM Positive and Negative Ion Calibration Solutions (Thermo Fisher Scientific). Acquired data were then converted from .raw to .mzXML file format using Mass Matrix (Cleveland, OH, USA). Metabolite assignments, isotopologue distributions, and correction for expected natural abundances of 13C isotopes were performed using MAVEN (Princeton, NJ, USA). Metabolite assignment was performed against an in-house standard library, as reported (Nemkov et al., 2015).

UHPLC-MS Lipidomics:
Commercial reagents were purchased from Sigma-Aldrich. (Saint Louis, MO) Approximately 100,000-200,000 ROS-Low LSCs were sorted, and analyses were performed via ultra-high pressure-liquid chromatography-mass spectrometry (UHPLC-MS – Vanquish and Q Exactive, Thermo Fisher, San Jose, CA, and Bremen, Germany) using C18 reversed-phase chromatography with separate runs in positive and negative mode, using electrospray ionization (ESI). Briefly, cells were extracted in ice-cold methanol at a concentration of 2 million cells/mL. Cells were briefly hand-vortexed then incubated without shaking at -20°C for 30 min. Samples were then centrifuged at 18,213g for 10 min at 4°C, and supernatants were diluted 1:1 with 10mM ammonium acetate in water. Ten microliters of sample extracts were loaded onto an Acquity HSS T3 column (150 × 2.1mm i.d., 1.8µm – Waters) at 45°C using a 17 min gradient at 300-400uL/min in negative mode. (25-99% B, A: 25% ACN, 75% water, 5mM ammonium acetate, B: 50% isopropanol, 45% ACN, 5% water, 5mM ammonium acetate) were used to elute metabolites. The mass spectrometer was scanned in Full MS mode or performed acquisition independent fragmentation (AIF - MS/MS analysis) at 70,000 resolution in the 150-1500 m/z range, 4 kV spray voltage, 45 sheath gas, and 15 auxiliary gas, operated in negative mode. Calibration was performed before analysis using the PierceTM Negative Ion Calibration Solution (Thermo Fisher

Scientific). Metabolite assignment was performed against an in-house standard library, as reported (Nemkov et al., 2015). Data obtained from MS/MS lipidomics method was analyzed using LipidSearch^{TM} (Thermo Fisher) to perform relative quantitation and metabolite assignment against the Thermo Fisher lipid database.

Metabolic Tracing:
200,000-500,000 ROS-Low LSCs were sorted and incubated with stable isotope substrates: 13C16-palmitic acid (Sigma-Aldrich, 705573), 13C6-glucose, and 13C18-linoleic acid (Cambridge Isotopes, CLM-6990-PK) for 1, 4, or 8hr. Glucose was added at 5.5mM. Palmitic acid and linoleic acid were added at 100µM. Media formulations remained consistent across all experiments, though each iteration contained a different single stable isotope-labeled tracer. From each independent experiment, we calculated the labeled isotopologues as a percentage of the total (labeled + unlabeled), normalized to the relative abundance of each substrate. Metabolomics analyses were performed using the 5 min method in Supplementary Methods.

Proteomic Mass Spectrometric Analysis
Samples were loaded onto a 1.5mm thick NuPAGE Bis-Tris 4−12% gradient gel (Invitrogen) and visualized with SimplyBlue™ SafeStain (Invitrogen) stain. Each lane of the gel was divided into 5 equal-sized bands, and proteins in the gel were digested as follows. Pieces were destained in 200µL of 25mM ammonium bicarbonate in 50% v/v ACN for 15 min and washed with 200µL 50% (v/v) ACN. Disulfide bonds were reduced by dithiothreitol, and cysteine residues were alkylated with iodoacetamide. Modified sequencing grade trypsin (Promega) was added 1:100 w/w and the samples were digested overnight at 37C. Digestion was stopped by the addition of 5% FA. Peptides were extracted two times from the gel plugs using 1% FA in 50% ACN. Collected extractions were pooled with the initial digestion supernatant, dried on a SpeedVac concentrator, and resuspended in 40uL 0.1 % FA. Each sample was loaded onto individual Evotips for desalting and then washed with 200µL 0.1% FA followed by the addition of 100µL storage solvent (0.1% FA) to keep Evotips wet until analysis. The Evosep One system ((Evosep, Odense, Denmark) was used to separate peptides on a Pepsep column (150 um inter diameter, 15 cm) packed with ReproSil C18 1.9 um, 120A resin. The system was coupled to the timsTOF Pro mass spectrometer (Bruker Daltonics, Bremen, Germany) via the nano-electrospray ion source (Captive Spray, Bruker Daltonics).
The mass spectrometer was operated in PASEF mode. The ramp time was set to 100ms and 10 PASEF MS/MS scans per topN acquisition cycle were acquired. MS and MS/MS spectra were recorded from m/z 100 to 1700. The ion mobility was scanned from 0.7 to 1.50 Vs/cm2. Precursors for data-dependent acquisition were isolated within ± 1 Th and fragmented with an ion mobility-dependent collision energy, which was linearly increased from 20 to 59 eV in positive mode. Low-abundance precursor ions with an intensity above a threshold of 500 counts but below a target value of 20000 counts were repeatedly scheduled and otherwise dynamically excluded for 0.4 min.

Database Searching and Protein Identification
MS/MS spectra were extracted from raw data files and converted to .mgf files using MS Convert (ProteoWizard, Ver. 3.0). Peptide spectral matching was performed with Mascot (Ver. 2.5) against the Uniprot human database. Mass tolerances were +/- 15 ppm for parent ions, and +/- 0.4 Da for fragment ions. Trypsin specificity was used, allowing for 1 missed cleavage. Met oxidation, protein N-terminal acetylation, and peptide N-terminal pyroglutamic acid formation were set as variable modifications with Cys carbamidomethylation set as a fixed modification. Scaffold (version 4.8, Proteome Software, Portland, OR, USA) was used to validate MS/MS-based peptide and protein identifications. Peptide identifications were accepted if they could be established at greater than 95.0% probability as specified by the Peptide Prophet algorithm. Protein identifications were accepted if they could be established at greater than 99.0% probability and contained at least two identified unique peptides.

Global UHPLC-MS Metabolomics, Lipidomics, and Metabolic Tracing

ROS-Low LSCs were enriched as described above and analyses were performed via UHPLC-MS, as extensively described in the Supplementary Methods. Metabolite assignment was performed against an in-house standard library, as reported (Nemkov et al., 2015). Data obtained from MS/MS lipidomics method was analyzed using LipidSearch^{TM} (Thermo Fisher.) Metabolic tracing was performed using U13C-glucose and U-13C-linoleic acid (Cambridge Isotope Laboratories).

Supplementary Table S1

	Sample Identity	Age	Sex	Blast %	Cytogenetics	Mutation notes
De novo 1	De novo	52	M		46,XY,add(1)(p11),del(5)(q15q33),del(7)(q22q36),der(11)t(1;11)(p31;p12-14)[20] , Loss of 5q31 and 7q31	FLT3 ITD, BCOR, NOTCH1
De novo 2	De novo	52	M		45,XY,-7[3]/46,sl,+r(7)(p11q21)[11]/46,sdl1,der(5)t(1;5)(q31;p14)[5]/46,XY[1]	ASXL1 c.4120_4121insT; DNMT3A c.885delG; Notch1 c.4746_4747insTGGGGA; NRAS c. 182A>G
De novo 3	De novo	49	F	65%	Normal karyotype (46, XX)	FLT3 ITD+; WT for CEBPA, NPM1, IDH1, IDH2, JAK2
De novo 4	De novo	73	M		46,XY[20]	
De novo 5	De novo					TET2 c.T5162G; p.L1721W (99.2%), NRAS c.G37C; p.G13R (30.2%), ASXL1 c.T2444C; p.L815P (99.8%), GATA2 c.T962A; p.L321H (52.0%), BCORL1 c.T331C; p.F111L(100%), BCORL1 c.C2912G; p.A971G (99.8%)
Relapse 1	Relapse	65	F	90%	46,XX,add(14)(q22)[4], 46,XX[16]	Mutant for FLT3, NPM1 and IDH1
Relapse 2	Relapse					WT for IDH1, IDH2
Relapse 3	Relapse	49	F	75%	Normal karyotype (46, XX)[21]	FLT3 ITD+ (50 myeloid panel or clinical PCR?)
Relapse 4	Relapse	47	M	97%	46,XY,del(7)(q21)[8]/46,sl,del(5)(q31q35),add(12)(p13)[7]/46,sl,add(12)(p13),del(17)(q21)[3]/46,XY,del(9)(q22q32)[2]	IDH1 R132; CKIT D816V; WT for IDH2, FLT3 and NPM1
Relapse 5	Relapse				46,XY[20]	NRAS.G13C 13%, GATA2.L375I 42%, KRAS.G12V 31%, CEBPA.P49Rfs*111 42%
Relapse 6	Relapse	36		98%	Normal karyotype	Mutant for FLT3, NPM1; WT for CEBPA, IDH1, IDH2

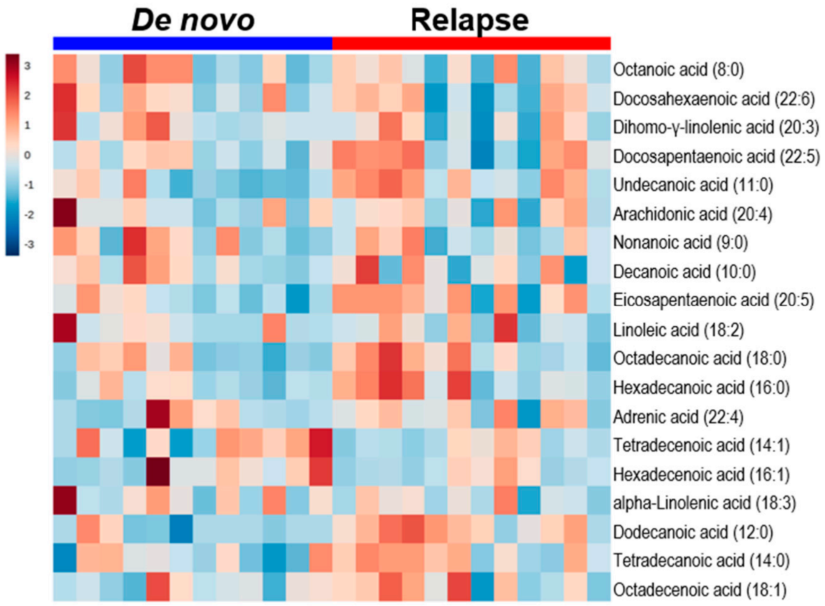

Supplementary Figure S1: Free fatty acid levels in AML leukemic blasts. Heatmap of free fatty acid levels in de novo and relapse AML leukemic blasts. Metabolomics analysis was performed with an n=3 patient samples with 4 technical replicates. (de novo patients 1, 2, and 3, and relapsed patients 1, 2, and 3.)

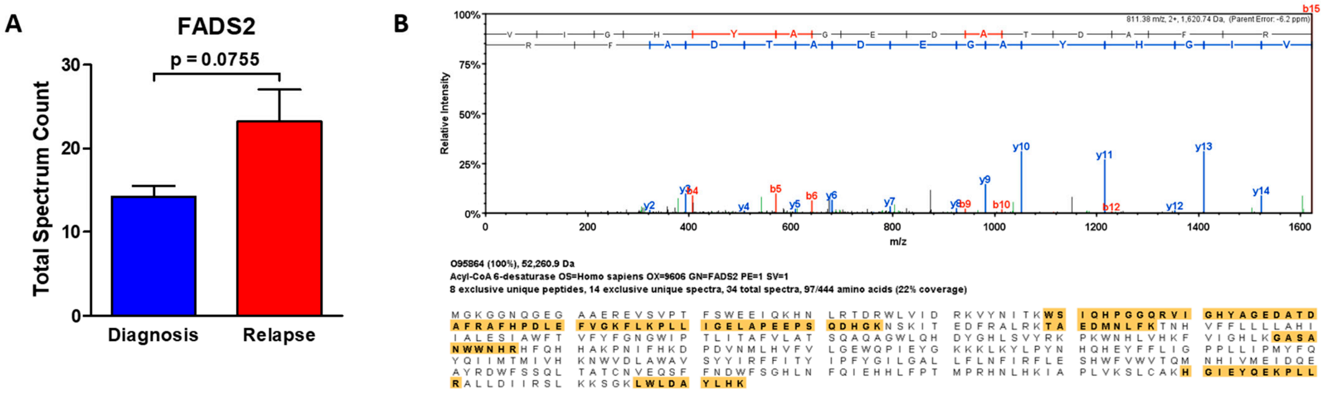

Supplementary Figure S2: Levels of FADS2 protein in AML stem cells. (A) Proteomic immunoprecipitation pulldown analysis of FADS2 (de novo patient 1, relapse patient 5). (B) Sequence coverage of FADS2.

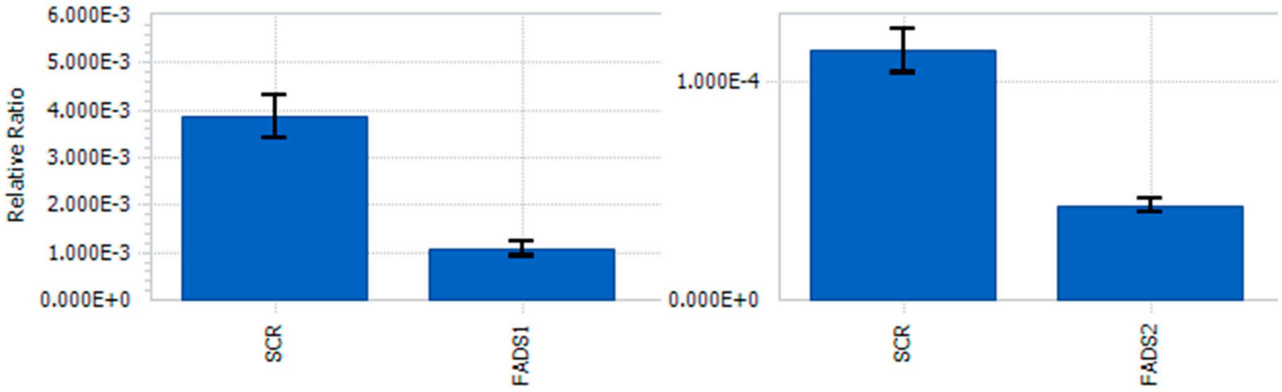

Supplementary Figure S3: Decreased expression of FADS1 and FADS2 upon siRNA knockdown. ΔΔCq expression of FADS1 and FADS2 after siRNA knockdown in MOLM-13 AML cells.

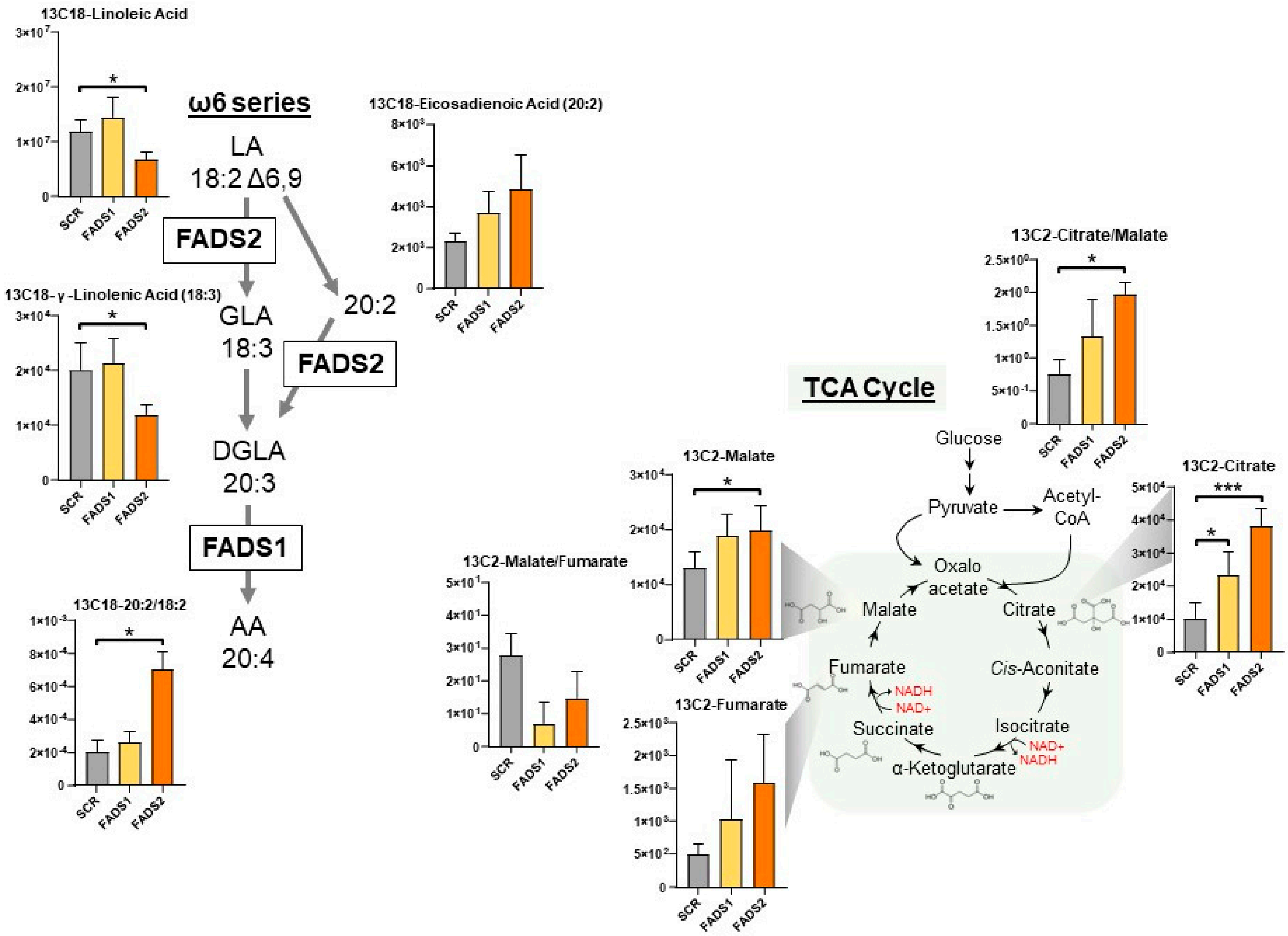

Supplementary Figure S4. Genetic inhibition of FADS1 and FADS2 decreases desaturation products of ω6 pathway but increases flux into the TCA cycle. Metabolic flux using stable heavy isotope labeled (13C-18) linoleic acid (18:2) at 8h post-treatment with siRNA KD of FADS1 and FADS2 in Molm13 cells.
* ≤ 0.05, ** ≤ 0.01, *** ≤ 0.001, **** ≤ 0.0001

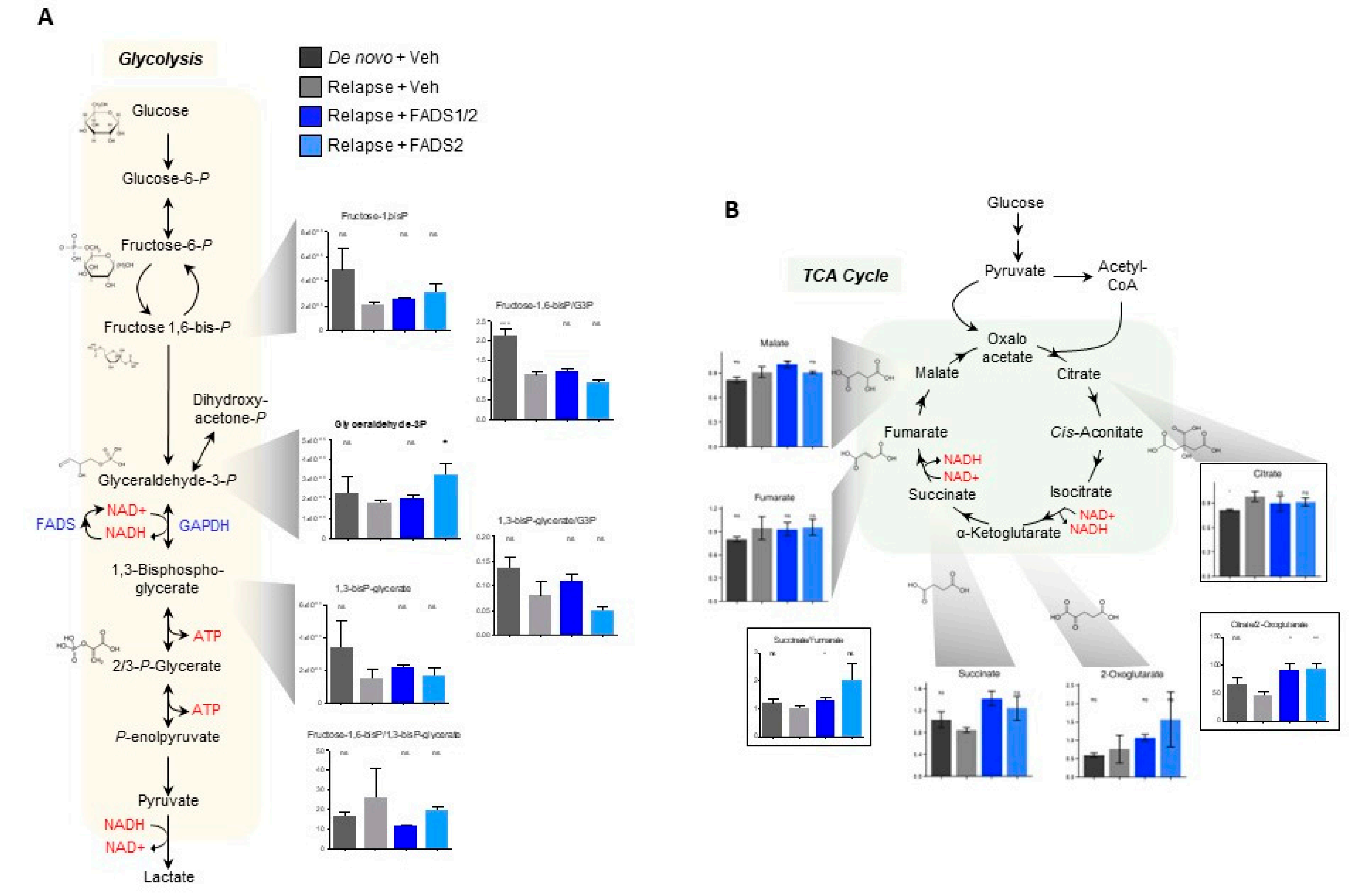

Supplementary Figure S5. Aberrant glycolytic activity in relapse LSC is reversed upon FADS inhibition. Relative abundance of glycolysis and TCA cycle intermediates in AML *de novo* LSCs, LSCs at relapse, and LSCs at relapse with inhibitors. Cells were incubated for 4h and levels were normalized to 1h. Performed in paired *de novo* and relapse patient 3 bulk patient AML cells.

* ≤ 0.05, ** ≤ 0.01, *** ≤ 0.001, **** ≤ 0.0001
